# Modeling Necroptotic and Pyroptotic Signaling in *Saccharomyces cerevisiae*

**DOI:** 10.3390/biom15040530

**Published:** 2025-04-04

**Authors:** Óscar Barbero-Úriz, Marta Valenti, María Molina, Teresa Fernández-Acero, Víctor J. Cid

**Affiliations:** Department of Microbiology and Parasitology, School of Pharmacy, Universidad Complutense de Madrid, Pza. de Ramón y Cajal s/n, 28040 Madrid, Spain; oscaralb@ucm.es (Ó.B.-Ú.); martva02@ucm.es (M.V.); molmifa@ucm.es (M.M.); teresafe@ucm.es (T.F.-A.)

**Keywords:** regulated cell death, humanized yeast, *Saccharomyces cerevisiae*, necrosome, inflammasome, caspases, pyroptosis, necroptosis, GSDMD, MLKL

## Abstract

The yeast *Saccharomyces cerevisiae* is the paradigm of a eukaryotic model organism. In virtue of a substantial degree of functional conservation, it has been extensively exploited to understand multiple aspects of the genetic, molecular, and cellular biology of human disease. Many aspects of cell signaling in cancer, aging, or metabolic diseases have been tackled in yeast. Here, we review the strategies undertaken throughout the years for the development of humanized yeast models to study regulated cell death (RCD) pathways in general, and specifically, those related to innate immunity and inflammation, with an emphasis on pyroptosis and necroptosis. Such pathways involve the assembly of distinct modular signaling complexes such as the inflammasome and the necrosome. Like other supramolecular organizing centers (SMOCs), such intricate molecular arrangements trigger the activity of enzymes, like caspases or protein kinases, culminating in the activation of lytic pore-forming final effectors, respectively, Gasdermin D (GSDMD) in pyroptosis and MLKL in necroptosis. Even though pathways related to those governing innate immunity and inflammation in mammals are missing in fungi, the heterologous expression of their components in the *S. cerevisiae* model provides a “cellular test tube” to readily study their properties and interactions, thus constituting a valuable tool for finding novel therapies.

## 1. Introduction: SMOC Assembly, Innate Immunity, and Cell Death

As a general concept, regulated cell death (RCD), as opposed to accidental cell death (ACD), integrates diverse intrinsic processes that multicellular organisms have evolved to utilize to induce cellular demise. RCD is essential to restore tissue homeostasis under stresses or aggressions, such as tumor development or the attack of pathogens [1,2]. The triggering of RCD pathways frequently results in the activation of caspases (CASPs), proteases directed to specific targets that, once proteolyzed, release a deadly executor. The clearance of damaged or infected cells is an effective strategy to block the spread of intracellular viruses or bacteria. Innate immunity and inflammation represent the first line of defense of our body against invading pathogens. Innate immunity involves a non-specific response mediated by pattern recognition receptors (PRRs) after their activation by cellular damage-associated molecular patterns (DAMPs)―endogenous danger signals―or pathogen-associated molecular patterns (PAMPs)―derived from microorganisms; typically, viruses, bacteria, or parasites. Toll-like receptors (TLRs), NOD-like receptors (NLRs), or RIG-I-like receptors (RLRs) are examples of PRRs involved in orchestrating a cellular response in response to PAMPs or DAMPs.

**Figure 1:**
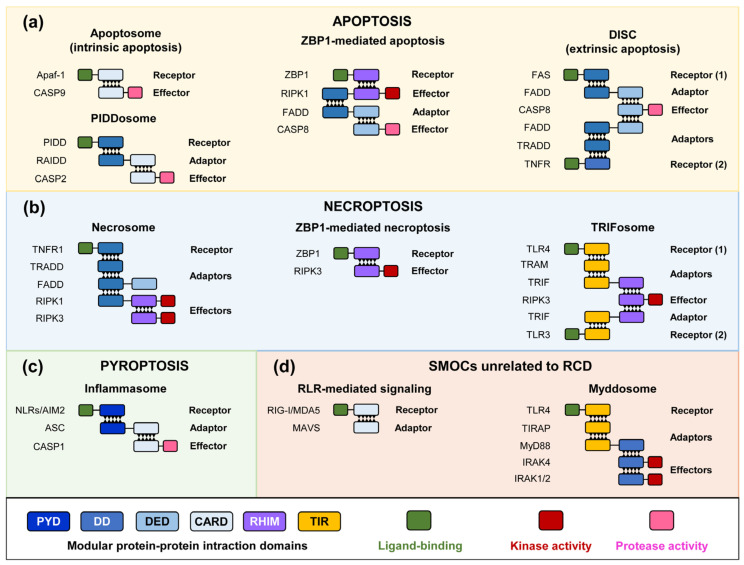
Simplified scheme of interactions mediated by typical modular domains that govern the assembly of key SMOCs in innate immunity and RCD. Signalosomes operating in apoptotic (**a**), necroptotic (**b**), and pyroptotic (**c**) cell death, together with innate immunity signaling complexes that show a similar architecture (**d**). Only single heterotypic interactions of core signalosomes are represented.

Unlike other signal transduction pathways that usually involve either a series of consecutive phosphorylation reactions upon receptor activation or the generation of second messengers, PRRs often signal through the assembly of supramolecular organizing centers (SMOCs) [3,4], a signaling mechanism that has been denominated Signaling by Cooperative Assembly Formation (SCAF) [5]. Most SMOCs follow a receptor–adaptor–effector hierarchic architecture. Once the receptors detect their respective ligands [bacterial lipopolysaccharide (LPS), flagellins, viral nucleic acids, etc.], they undergo conformational changes that trigger their oligomerization. Multimeric receptors serve as a nucleating platform for adaptor proteins, which eventually recruit the corresponding enzymatic effectors. In turn, such effectors become active by proximity-induced mechanisms [6]. In most SMOCs, the recruitment of the different receptor, adaptor, and effector components is driven by homotypic interactions between protein–protein interaction domains. Six families of these domains have been described: DDs (death domains), DEDs (death–effector domains), CARDs (caspase recruitment domains), PYDs (pyrin domains), TIR (Toll-interleukin-1 receptor) domains, and RHIMs (receptor-interacting protein homotypic interaction motifs) (Figure 1). The first four belong to the DD superfamily and display a similar six-helix bundle fold, but the differences in sequence and structure confer them specificity [7,8]. SMOCs help to amplify the signal in an all-or-none response, increase the sensitivity of the immune response, and modulate its strength [3]. Thus, RCD is the outcome of SMOC assembly in the presence of diverse stimuli, many related to innate immunity but also to diverse stress responses. In this review, we will describe the signal pathways leading to the activation of the most common forms of RCD and the general features of the proteins provoking cell disruption downstream of these pathways and focus on the contribution that the eukaryotic model *Saccharomyces cerevisiae* has made to this field.

## 2. RCD Pathways and SMOC Assembly

Among the diverse processes cataloged as RCD, apoptosis, necroptosis, and pyroptosis are the most studied, although several other RCD processes have been recently delineated [2].

Apoptosis is an RCD process defined by phosphatidylserine exposure and certain morphological features such as membrane blebbing, cell shrinking, and nuclear condensation [9]. Because the plasma membrane is retained, it is generally considered a non-inflammatory mechanism of RCD. Apoptosis depends on the function of particular types of effectors that are cysteine–aspartic proteases (caspases) that hydrolyze intracellular substrates after an aspartic residue is allocated in a particular sequence and carry a crucial cysteine residue in their active center. They usually contain specific domains at their N-termini that allow the interaction with pre-assembled components of the SMOC. Caspases are zymogens synthesized as pro-caspases that require proteolysis either by other proteases or by themselves for their full catalytic activation. Apoptotic caspases are categorized into initiators (caspases 2, 8, 9, and 10), recruited to the SMOCs and self-activated, and executioners (caspases 3, 6, and 7), activated by initiator-dependent cleavage [10]. There are two pathways leading to apoptosis depending on the location of the trigger stimulus. Extrinsic apoptosis is triggered by extracellular ligand binding to receptors such as the FS-7 cell line-associated antigen (Fas) or tumor necrosis factor receptor (TNFR). The anchorage of the adaptors Fas-associated death domain (FADD) and TNFR1-associated death domain (TRADD), respectively, drives the recruitment of pro-caspase-8, resulting in a SMOC named the death-inducing signaling complex (DISC), constituted by interactions between DED and DD domains (Figure 1). The formation of this complex leads to the activation of caspase-8, provoking the cleavage and activation of the executioner caspase-3 and caspase-7 that produce cell death through the processing of a large number of intracellular substrates [11,12].

In contrast, intrinsic apoptosis occurs upon mitochondrial damage. BCL2 family members, through the formation of pores within the mitochondrial outer membrane, provoke the release of cytochrome c, which triggers the recruitment of the apoptotic protease-activating factor 1 (Apaf-1) and the assembly of the apoptosome (Figure 1). This complex relies on interactions between the CARD domains of Apaf-1 and caspase-9, which become activated in the apoptosome and subsequently process the executioner caspases-3 and -7. An alternative SMOC called PIDDosome can also activate apoptosis. This complex is assembled by the interaction of the p53-induced death domain (PIDD1) protein, the RIP-associated ICH-l/CED-3-homologous protein with a death domain (RAIDD) adaptor, and caspase-2, which is activated upon PIDDosome binding (Figure 1). Caspase-2 can induce apoptosis through the processing of BID, which triggers intrinsic apoptosis but is also linked to cell differentiation, cell cycle regulation, genotoxic stress-induced RCD, and tumor suppression [13,14].

Pyroptosis is a lytic RCD process that typically occurs in infection scenarios and ends with the formation of plasma membrane pores, through which cytokines and other molecules are released from the cell, causing the activation of an inflammatory response. In addition to cell rupture, pyroptotic cells exhibit nuclear condensation and cell swelling. The canonical SMOC regulating pyroptosis is called the inflammasome [15]. It is composed of an innate immune sensor containing a pyrin domain (PYD), such as the NOD-like receptors (NLRs) or AIM2-like receptors that, upon PAMP or DAMP detection, form a complex with the adaptor ASC (apoptosis-associated Speck-like protein containing a CARD) through PYD–PYD domain interactions. The CARD domain in ASC recruits, in turn, the inflammatory caspase-1 through CARD–CARD interactions (Figure 1). The effector of the inflammasome is the pore-forming protein Gasdermin D (GSDMD), requiring directed proteolytic cleavage by active caspase-1 to release its pore-forming subunit. The most significant outcome of pyroptosis is the massive release of interleukin 1β (IL-1β), which itself relies on caspase-1 activity for proteolytic maturation, but also a variety of intracellular molecules acting as DAMPS for the immune system [16].

Necroptosis is a lytic and inflammatory form of RCD that exhibits morphological features of both necrosis and apoptosis, such as cell swelling and plasma membrane rupture [17,18]. It occurs in response to inflammation and infection and can be induced by several receptors. One of them, TNFR1, when stimulated by TNF binding, activates several pathways. On one hand, it activates the transcription factor NF-κB in order to promote inflammation. It also provokes apoptosis by the activation of caspase-8 via the adaptor FADD. In addition, when caspase-8 is pharmacologically inhibited [19,20] or targeted by certain viruses [21], a SMOC named necrosome is assembled. Although this signaling complex has not been considered a SMOC, it is known to assemble amyloid complexes [22]. The adaptors TRADD and FADD interact with TNFR1 and with receptor-interacting serine/threonine protein kinase-1 (RIPK1), promoting its autophosphorylation, which in turn recruits RIPK3 by RHIM–RHIM domain docking (Figure 1). RIPKs represent the key effectors of this pathway since their elimination impedes the assembly of the necrosome and triggers apoptosis. Indeed, when the apoptosis response is started, RIPK1 is cleaved by caspase-8, leading to the inactivation of necroptosis [23]. The autophosphorylation of RIPK3 promotes the phosphorylation and activation of the mixed lineage kinase domain-like (MLKL) pseudokinase, the lytic executor of the pathway [24,25]. In addition, RIPK3 can also be activated upon viral DNA recognition by the Z-DNA-binding protein 1 (ZBP1) sensor, which directly binds to RIPK3 through its RHIM domain [26], triggering ZBP1-mediated necroptosis (Figure 1).

Necroptosis can also be activated through the classical innate immune receptors TLR4 and TLR3. These receptors primarily participate in the assembly of the myddosome, a key complex transducing PAMP detection [27]. The myddosome assembly starts after the interaction of the dimerized TLR with the TIR-containing adaptors TIRAP and MyD88 at the PM (Figure 1) or TRAM and TRIF at the endosome, all mediated by TIR–TIR interactions. This allows the docking of the IL-1 receptor-associated kinase 4 (IRAK4) protein kinase and, subsequently, both IRAK1 and IRAK2. These kinases phosphorylate the ubiquitin ligase TRAF6, which is fundamental for cytokine and chemokine gene expression [28]. The myddosome is linked to pyroptosis since the transcriptional activation of inflammasome components, like NLRP3, is triggered by TLR signaling. However, IRAK1 can bypass protein synthesis and activate the inflammasome directly [29]. Both TLR3 and TLR4 interact either directly in the case of the former or through the adaptor TRAM in the case of the latter via TRIF with RIPK3 (Figure 1) and are able to trigger necroptosis when caspase-8 is inhibited [30].

Although apoptosis, pyroptosis, and necroptosis operate linearly, multiple crosstalks have been described among them, giving rise to a new type of RCD called PANoptosis (P, pyroptosis; A, apoptosis; N, necroptosis), which shares components and possesses features common to the three of them [31,32,33,34]. PANoptosis has been reported in response to infections but also in complex pathologies like cancer and neurodegenerative and inflammatory diseases [35]. Four types of PANoptosomes have been described so far: the ZBP1 PANoptosome, the AIM2 PANoptosome, the RIPK1 PANoptosome, and the NLRP12 PANoptosome [36]. Thus, PANoptosomes involve SMOC components of all three main RCD processes, provoking cell death when other cell defenses are blocked.

There are still innumerable open questions about the complex quaternary structure of SMOCs, the hierarchy of their assembly, the attachment of SMOC components to specific landmarks in the cytoplasm, the functional redundancies among their molecular components, and the intricate crosstalk among multiple RCD and innate immune pathways. All these questions demand new investigative approaches. Among the diverse experimental settings to gain insight into these topics, in Section 4, we highlight heterologous expression in the budding yeast model *S. cerevisiae*.

## 3. Pore-Forming Executors of Cell Death: Pyroptosis vs. Necroptosis

**Figure 2:**
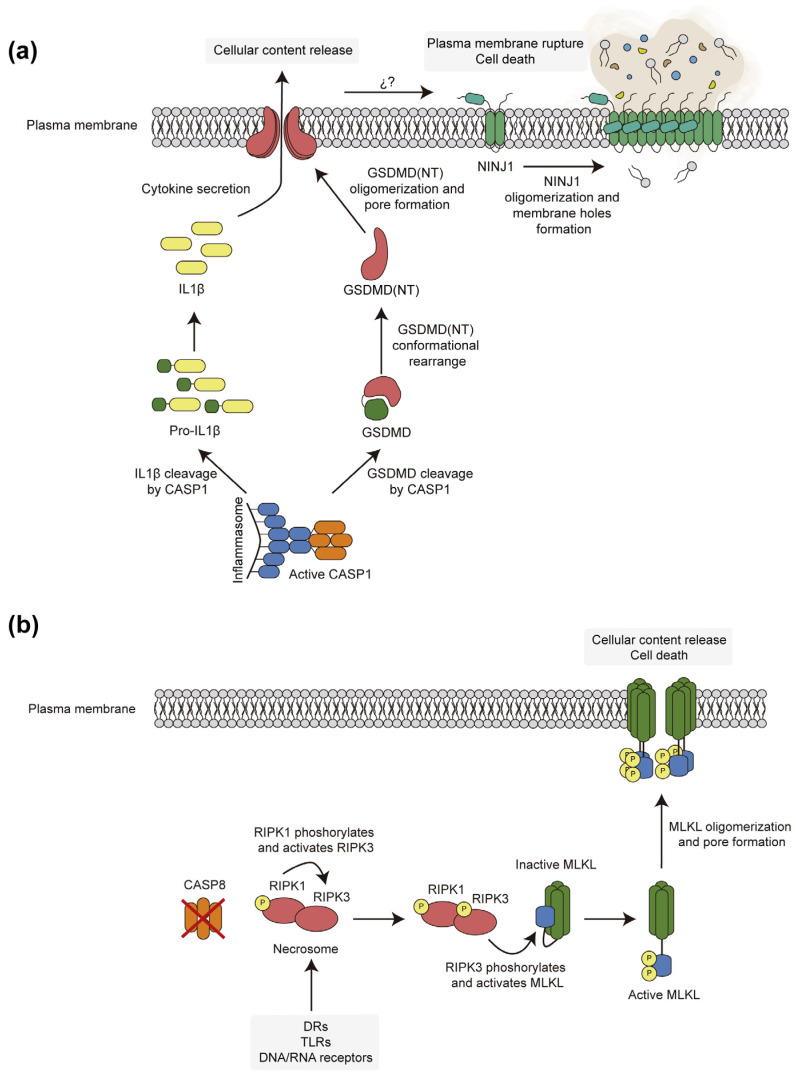
Schematic representation of pyroptotic (**a**) and necroptotic (**b**) pathways. (**a**) In pyroptotic RCD, inflammasome-mediated activation of caspase-1 (CASP1) leads to processing of both pro-IL-1β and GDSMD. The latter releases its N-terminal domains (NT) to assemble pores that facilitate the release of processed pro-inflammatory IL-1β and causes membrane integrity and ion permeability changes that eventually trigger NINJ1 polymerization to promote dramatic plasma membrane rupture. (**b**) In the absence of caspase-8 (CASP8) activity, RIPK kinases respond to death receptors (DRs) or other stimuli to promote the activation of the MLKL pore as a consequence of conformational changes induced by interaction and phosphorylation. These pores destabilize membrane homeostasis to cause necroptosis but do not seem to stimulate NINJ1-mediated plasma membrane rupture.

As mentioned, the lytic processes of RCD, namely pyroptosis and necroptosis, involve cell rupture through different executors: GSDMD in pyroptosis and MLKL pseudokinase in necroptosis. Figure 2 provides a bird’s eye comparative view of both processes.

GSDMD was the first member of the Gasdermin (GSDM) family to be related to pyroptosis [37,38], but the family comprises six proteins in human cells: Gasdermin A (GSDMA), Gasdermin B (GSDMB), Gasdermin C (GSDMC), GSDMD, Gasdermin E (GSDME) or DFNA5 (non-syndromic neurosensory deafness autosomal dominant type 5), and Gasdermin F or PJVK (Pejvakin). Despite differential tissue expression, they share a similar structure, activity, and activation mechanism, except for PJVK [39]. All GSDMs comprise a pore-forming N-terminal domain and an autoinhibitory C-terminal domain. The latter domain keeps the former in an inactive conformation under resting conditions. Its removal by proteolysis at the linker between both domains allows the conformational rearrangement of the N-terminal domain, which in turn oligomerizes and interacts with cellular membranes. Notably, the assembly of GSDM pores within the plasma membrane eventually leads to the release of cellular contents and cell death. The proteases that activate each family member are specific and determine their function [40,41]. CASP-1 cleaves GSDMD at D275 to release its N-terminal domain GSDMD (NT) [37,42,43], but, alternatively, GSDMD can also be activated by mouse caspase-11 or its human homologs caspase-4 and -5, other pro-inflammatory caspases that sense cytosolic LPS and form a non-canonical inflammasome, constituted only by the protease [44,45]. The conformational change in the released GSDMD N-terminal domain promotes interaction through a positively charged region, with negatively charged lipids present in the inner layer of the plasma membrane, primarily phosphatidylserine and phosphoinositides, such as phosphatidylinositol 4-phosphate [PI(4)P] and phosphatidylinositol 4,5-*bis*phosphate [PI(4,5)P_2_] [39,46]. Thirty-three GSDMD (NT) oligomers are assembled into ring-shaped structures of about 20 nm in inner diameter that insert into the plasma membrane to form non-selective pores [47,48]. It has been recently found that S-palmitolylation at Cys191 is essential for its pore-forming activity [49,50], as evidenced by the inhibitory effect of disulfiram, which is exerted by blocking this residue [51]. Although such pores are allegedly responsible for the rupture of the plasma membrane leading to pyroptotic RCD, this view has now been challenged by recent studies that suggest that GSDMD (NT) induces plasma membrane disruption via ninjurin-1 (NINJ1), a small transmembrane protein also involved in cell adhesion processes [52,53]. Just after the assembly of GSDMD (NT) pores within the plasma membrane, NINJ1 forms filaments that eventually trigger membrane dysfunction and rupture (Figure 2a) [54].

The necrosome serves as a platform to phosphorylate and thus activate the lytic protein MLKL, whose presence in the cell is essential for necroptotic cell death. MLKL is differentially expressed depending on the tissue, with the immune system and the bone marrow showing the highest expression [55]. MLKL contains 469 residues forming an N-terminal four-helical bundle domain (4HBD) and a C-terminal pseudokinase domain (PSKD) connected by two brace helices. PSKD can reside in an open–inactive or closed–active conformation. In resting conditions, the open form of MLKL forms a stable heterodimer with RIPK3 in which the PSKD represses the activity of the 4HB domain. In human cells, upon necrosome formation, RIPK3 phosphorylates the PSKD at T357 and S358 sites [56]. This causes the release of the heterodimer and the transition to the closed–active conformation. The model of MLKL oligomerization and the precise stoichiometry of the oligomeric structure is still being investigated, with claims to form dimers [57], trimers [58], tetramers [59], and hexamers [56]. The two-brace helices seem to play an important role in the assembly of the oligomers but also in interdomain communication [60]. Oligomerized MLKL traffics to the plasma membrane via Golgi vesicles involving the actin and microtubule cytoskeletons, where it interacts with phospholipids and phosphoinositides through electrostatic interactions with positive residues within the 4HB domain [61,62]. The activity of MLKL alters the ion fluxes across the plasma membrane and provokes the release of cellular contents, leading to cell death. Several models have been proposed for MLKL-based cell disruption, such as the formation of pores [63] or a prion-like action of the oligomers inserted at the PM [64]. The structural resolution of MLKL oligomers within the plasma membrane revealed differences from that of GSDMD (NT) pores [61,65]. In contrast to the latter, MLKL can remain at the plasma membrane for a longer period of time than GSDMD and induces a more limited cell permeability [61,63]. These differences could set the basis for the different kinetics of death and the morphological features of pyroptotic and necroptotic cells. Indeed, unlike in the case of GSDMD, the role of NINJ1 in MLKL-driven plasma membrane rupture seems to be negligible [53]. In addition to plasma membrane rupture, MLKL regulates other cellular processes. RIPK3-activated MLKL counteracts its own lytic activity by enhancing its trafficking to the extracellular space [66,67] or its endocytosis [68]. Interestingly, the ESCRT-III machinery complexes maintain membrane integrity while MLKL is active, thus delaying cell death [69]. MLKL can also trigger inflammasome activation through the alteration of ion fluxes [70] and can act as a transcriptional regulator by entering the nucleus through its NLS [71,72].

Both GSDMD and MLKL are important for eliciting responses against pathogens, but their deregulation is linked to several diseases [73]. Thus, nowadays, they are considered promising drug targets in the fields of autoimmunity, inflammation, and cancer.

## 4. Modeling SMOC Assembly, Necroptosis, and Pyroptosis in Yeast

### 4.1. Heterologous Expression of Caspases in S. cerevisae

Although the budding yeast genome encodes a unique member of the functionally related metacaspase family, named Mca1 or Yca1 [74,75], the yeast proteome essentially lacks canonical caspases. However, caspase-3-, caspase-6-, and caspase-8-like activities have been biochemically detected [76]. In response to diverse challenges, especially those causing oxidative stress or killer toxins, *S. cerevisiae* exhibits cell death patterns with traits reminiscent of those of apoptosis in vertebrates. These include nuclear fragmentation, chromatin condensation, altered mitochondrial potential, cytochrome c release, or phosphatidylserine exposure at the outer leaflet of the plasma membrane. Furthermore, yeast proteins with domains of limited structural similarity to caspases have been described, namely Gpi8 [77] and the separase Esp1. The latter protein was reported to be involved in an apoptosis-like behavior in yeast cells [78]. Some of these RCD-like features require the metacaspase Yca1, but a proper apoptotic pathway resembling that of higher cells has not been traced in yeast to date. Nevertheless, *S. cerevisiae* has proved a suitable model to study metazoan caspases by heterologous expression, based on the fact that the overproduction of their active forms inhibits yeast growth [79,80].

In 1999, three different research groups pioneered the use of *S. cerevisiae* as a model to study caspase function by expressing both human and *Drosophila* caspases [81,82,83]. Hawkins et al. devised a reporter system for caspase activity in yeast based on the proteolytic release of a transcriptional activator bound to a transmembrane protein by a caspase target sequence [82]. In their report, they noted that active caspases were toxic for yeast in a dose-dependent manner. Thus, it was soon observed that initiator pro-caspases were self-activated in yeast and able to activate downstream executioner caspases in turn. The latter were generally non-toxic when expressed as pro-caspases but severely inhibited growth if truncated forms mimicking their proteolytic activation were produced. Kang et al. [81] first reported that initiator pro-caspases caspase-8 and caspase-10 were able to auto-activate in yeast and to activate executioner caspase-3, but not caspase-6, providing clues on the hierarchy of initiator–executor pathways. Later, the Hawkins lab, also using yeast, showed that executioner caspase-7 could be activated by caspase-2, although to a lesser extent than by its purported initiator caspase-9 [84]. Indeed, growth inhibition has been the main readout to study heterologous caspase activity in this heterologous model [79,80], and the basis for assaying caspase inhibitors. Co-expressed caspase antagonistic proteins, such as the baculovirus protein p35 or the mammalian inhibitors of apoptosis (IAPs), as well as chemical caspase inhibitors, have been successfully tested in the yeast model (Table 1). The system has additionally been exploited to assess the function of pro-apoptotic antagonists of IAPs, like DIABLO/Smac [85], or even to test caspase-activating compounds [86]. Furthermore, yeast has been engineered to study the substrate specificity of mammalian caspase-2 [87].

The terminal phenotype induced by caspase-8 production in *S. cerevisiae* shows some signs of apoptosis, such as annexin V externalization and ROS synthesis, but does not rely on yeast apoptotic machinery (i.e., Yca1, Aif1, and mitochondria) [79,80,96]. This has allowed designing yeast-based assays to test the activation, inhibition, specificity, or different properties of caspases [82].

**Figure 3:**
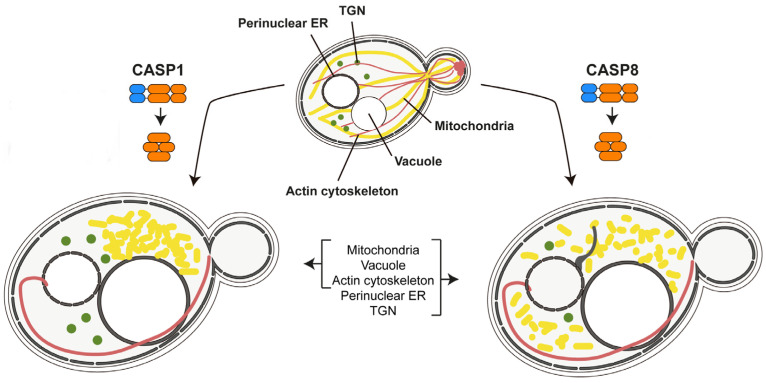
Effects on yeast organelles as a consequence of human caspase-1 (CASP1) and caspase-8 (CASP8) expression [99]. Both caspases were able to auto-activate in the yeast cell, leading to diverse observable effects in organelle size and distribution, as well as in the actin cytoskeleton, ultimately causing severe toxicity. The alteration of the endoplasmic reticulum (ER) and Golgi (TGN, trans Golgi network) were only observed for caspase-8.

The precise mechanisms by which caspases interfere with yeast biology have not been studied in depth. Although caspase-3 can replace the yeast Yca1 function in dissolving protein aggregates [97], it is generally accepted that spurious cleavage of yeast essential proteins is the most likely cause of their severe toxicity. Puryer and Hawkins noted that caspase-3 and caspase-8 disrupted cellular membranes without causing DNA damage, and that endogenous metacaspase Yca1 was not involved in heterologous caspase-induced effects in the yeast cell [80]. The toxicity of caspase-10 was also reported to be Yca1-independent and characterized by alterations in the endoplasmic reticulum and vacuolar morphology, as well as actin cytoskeleton disorganization [96]. These effects were suppressed by the deletion of genes coding for the protein kinases of the MAPK family, protein phosphatases, and the proteins of the factor-arrest (Far) protein complex at the Golgi, revealing a multifaceted effect on yeast physiology [98]. When the effects of caspase-1 and caspase-8 in the yeast cell were compared, the latter showed a more dramatic effect. Both caused mitochondrial fission, accompanied by condensation in the case of caspase-1, a disruption of actin structures, which reassembled as a single large cytoplasmic filament, and vacuole enlargement. Additionally, caspase-8 also affected the morphology of the endoplasmic reticulum and the abundance of Golgi spots (Figure 3) [99]. Like previously tested mammalian caspases, the effects of caspase-1, including mitochondrial hyperpolarization, were Yca1-independent. The Bni1 formin, essential for the proper assembly of the actin cytoskeleton that supports polarized secretion for budding, was detected as a proteolytic target for the heterologous caspase [99].

### 4.2. Yeast-Based Models for Inflammasome and Necrosome Assembly

Heterologous gene expression in yeast has been successful in establishing models for the study of the mitochondrial apoptotic pathway, since mammalian Bax, the canonical pro-apoptotic pore-forming protein belonging to the Bcl-2 family, induces cytochrome c release and yeast cell death. However, although Bax-induced cell death shares some, but not all, features of mammalian apoptosis, cytochrome c release is dispensable for this effect [100,101,102]. Importantly, the lethal effect of Bax can be suppressed by the co-expression of anti-apoptotic members of the same family, notably Bcl-2 [103]. However, yeast lacks the apoptosome, consisting of Apaf-1 and caspase-9, which triggers eventual executor caspase-3 activation in mammalian cells in response to cytochrome c release. Hawkins and co-workers [85] were able to reconstitute an active apoptosome in yeast by co-expressing pro-caspase-9 and pro-caspase-3 with a constitutively active version of Apaf-1 generated by deleting its WD-40 domains (Apaf-1^530^) [104]. In this setting, the co-expression of pro-caspase-9 and pro-caspase-3 was not toxic, but the expression of these caspases together with the constitutively active Apaf-1^530^ construct was [85]. This elegant experimental setting allowed for the testing of fly and human IAPs in the yeast heterologous model.

The assembly of the heterologous signaling complexes upstream caspases is a challenge due to the above-described autoprocessing capacity of these proteases when produced in yeast. Successful yeast-based models to study such SMOCs require titrating very carefully the expression levels of caspases to achieve sublethal intracellular amounts, so that their activation by upstream receptor–adaptor complexes can be achieved. The use of strong inducible promoters, like pGAL1, widely used for heterologous expression in yeast, is discouraged, although it is feasible to fine-tune induction to sublethal levels by using glucose/galactose ratios for caspase-1 [99]. Using combinations of yeast promoters of different strength and high copy vs. low copy expression vectors, Hiyashi and co-workers [105] were able to functionally reconstitute several SMOCs. These authors devised an engineered yeast platform in which caspase sequence targets linked a membrane trap to a transcription factor, so that transcriptional readouts such as β-galactosidase or auxotrophic markers would only be expressed upon caspase activation. By these means, the individual expression of all 10 human caspases allowed the testing of their specificity for diverse (W/D/T/L)-E-(H/V/T)-D protease target signals. Moreover, binary combinations of adaptor–effector were tested, successfully leading to the activation of pro-caspase-1 expressed from the weak constitutive promoter pCYC1 by ASC, pro-caspase-2 by RAIDD, pro-caspase-8 and pro-caspase-10 by FADD, and pro-caspase-9 by Apaf-1^530^. Furthermore, the same authors were able to reconstitute functional ternary complexes for the DISC and the inflammasome. For the former, the expression of Fas allowed the activation of both pro-caspase-8 and pro-caspase-10 only when FADD was present at low levels. For inflammasome reconstruction, the NLRP3 receptor deleted its Leu-rich repeats (NLRP3ΔLRR), and thus, constitutively active, expressed from strong constitutive pTEF or inducible pGAL1 promoters, was able to activate pro-caspase-1 specifically in the presence of the ASC adaptor, both expressed from weak promoters. NLRP1ΔLRR, however, was sufficient for pro-caspase-1 activation in the absence of ASC, recapitulating results in higher cells [105]. The pioneering and comprehensive work by Hiyashi et al. proves the applicability and versatility of the yeast model to screen for SMOC inhibitors or to functionally assay mutations in inflammasome components.

GSDMD and GSDME have also been expressed and functionally studied in the yeast model. GSDMD and GSDME N-terminal parts [GSDMD/E(NT)], as released by caspase activity, led to severe growth arrest when produced by themselves in yeast, whereas full-length Gasdermins did not [106,107]. This underscores the autoinhibitory role of GSDMD and GSDME C-terminal extensions and provides a yeast-based platform to assay or screen for putative Gasdermin agonists. These are recently described small molecules that bypass the need for inflammasome-dependent caspase-1 activation and may have therapeutic applications [108]. We found that the co-expression of human caspase-1 and GSDMD in yeast led to cleavage of the latter, thus reproducing the key molecular event leading to pyroptotic cell death [99]. Furthermore, Ji and Hawkins systematically tested the activity of several caspases in yeast and found that caspase-1, -4, -5, and -8 expression led to enhanced toxicity in yeast in the presence of GSDMD, whereas GSDME was processed by caspase-1, -2, and -3 [107]. These authors proved that the pan-caspase inhibitor Q-VD-OPh successfully reverted lethal phenotypes of the caspase–Gasdermin combinations, proving the yeast model useful for pharmacologic studies.

As explained above, the roles of MLKL seem to be more complex than sheer pore formation in the plasma membrane. The reconstitution of the necrosome in yeast can provide a valuable model to study the molecular interactions that drive necroptosis in higher cells. This approach was successfully undertaken by Ji et al., who expressed and functionally assessed the human necrosome components both individually and as a whole [109]. In their experimental setting, neither human MLKL nor RIPK1 was toxic for yeast cells. Nevertheless, either RIPK3 or mouse MLKL inhibited yeast growth, showing a difference between both MLKL orthologs. In addition, the co-expression of murine RIPK3 and MLKL provoked MLKL phosphorylation, leading to increased toxicity in yeast cells. This proved its suitability for testing the modulators of necrosome assembly and function [109]. Furthermore, we observed that the expression of the N-terminal 4HB domain of human MLKL in *S. cerevisiae*, but not full-length MLKL, was toxic for yeast cells, which reinforces the importance of this region in MLKL activity and the regulatory role of the C-terminal region [106].

### 4.3. Necroptotic and Pyroptotic Pore-Forming Cell Death Executors Kill Yeast Cells by Means Other than Cell Lysis

Permeability to propidium iodide has been used as a readout for the loss of plasma membrane selective permeability and the loss of cell integrity in both yeast and higher cells, being a standard assay for necroptotic and pyroptotic cell lysis. However, when pore-forming GSDMD (NT) and MLKL were endogenously expressed in yeast, cell lysis did not accurately correlate with their severe toxicity [106]. The overproduction of both full-length human MLKL and its 4HB domains alone in yeast led to defects in endocytic traffic, even though only the latter form was severely toxic [106]. As mentioned above, MLKL is known to modulate intracellular trafficking to provide support for the formation of intraluminal and extracellular vesicles in mammalian cells. Phosphomimetic MLKL T357E/S358D or other mutants mimicking structural changes derived from RIPK3-dependent phosphorylation enhanced extracellular vesicle generation [66]. However, in the yeast system, human MLKL phosphomimetic mutants did not behave significantly differently as compared to the wild type, regarding both toxicity and interference with vesicular traffic [106]. Thus, even in the absence of other regulatory inputs, studies in the yeast model may provide clues about the intrinsic ability of pore-forming proteins to interfere with intracellular trafficking events.

**Figure 4:**
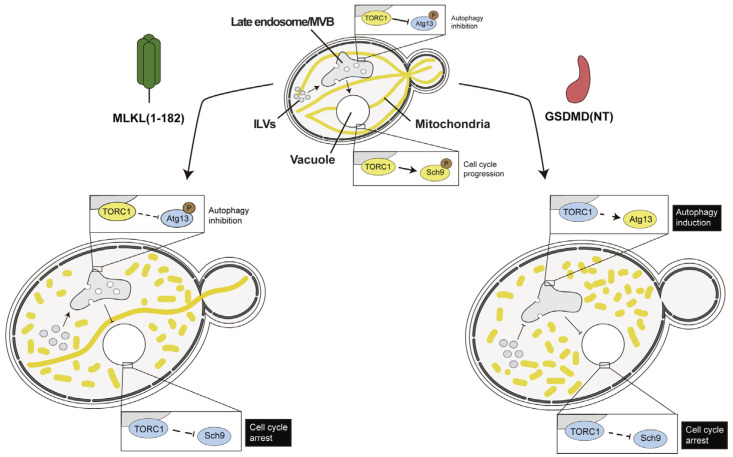
Effects of MLKL and GSDMD pore-forming fragments in yeast. The phenotypes observed upon heterologous expression of truncated MLKL (1-182) and GSDMD (NT) were very similar, involving mitochondrial fragmentation and the disruption of endocytosis. These effects led to TORC1 inhibition and cell cycle arrest. TORC1 kinase downregulation was evidenced by altered phosphorylation of its target, the Sch9 protein kinase and, in the case of GSDMD (NT), Atg13, involved in autophagy. However, TORC1 inhibition did not lead in any case to the expected triggering of autophagy, likely due to GSDMD (NT) and MLKL interference with trafficking events downstream. All observed effects were more severe in GSDMD (NT) than in full-length or truncated MLKL. Remarkably, GSDMD (NT) inhibited endocytosis at the stage of formation of multivesicular bodies, whereas MLKL blocked the fusion of late endosomes with the vacuole (equivalent to lysosomes in higher cells) [106]. MVB, multivesicular bodies; ILV, intraluminal vesicles.

Peculiarly, GSDMD toxicity in yeast was based on cell cycle arrest rather than on cell lysis [106]. As shown in Figure 4, we determined that GSDMD (NT) interfered with yeast endocytic traffic more severely than MLKL, leading to a dysfunction of the target of Rapamycin complex 1 (TORC1), which triggers growth arrest, as assessed by increased Sch9 phosphorylation [106]. This is intriguing, as pyroptosis-independent interleukin secretion related to the interaction of GSDMD with trafficking regulators has been described as an alternative function of GSDMD during inflammation, but this relies on interactions driven by its C-terminus rather than on its N-terminal pore-forming fraction [110]. Also, in macrophages, the lysosomal Ragulator complex and mTORC1 activity are essential for GSDMD pore formation in pyroptosis [111]. Thus, although many loose ends still need to be tied up, evidence from both higher cells and yeast relates GSDMD to interference with endosomal membranes and metabolic regulation.

### 4.4. ASC and Other SMOC Adaptor Proteins in Yeast: A Prion-Like Model

**Figure 5:**
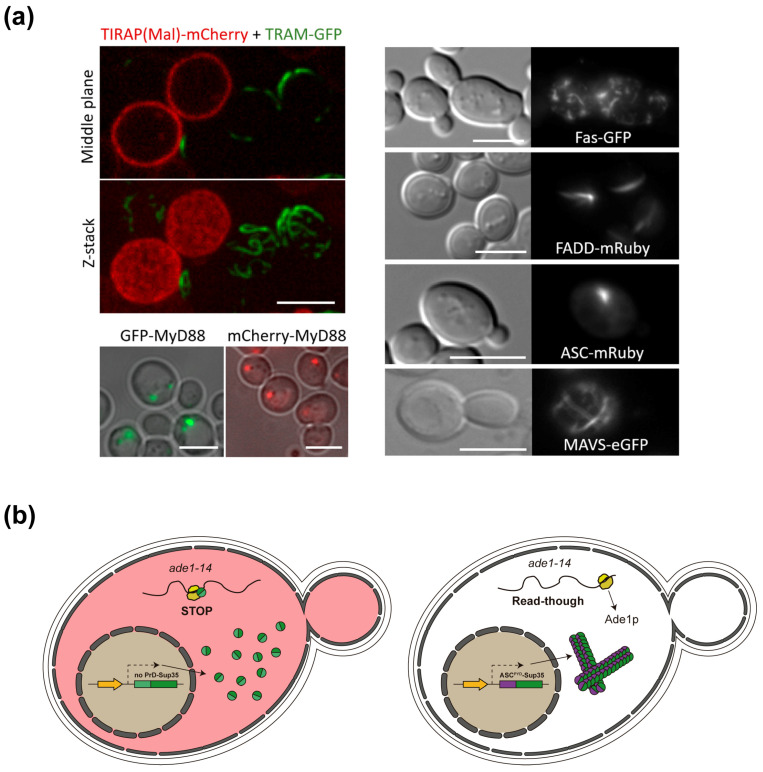
Aggregative and prionic properties of SMOC adaptors in yeast. (**a**) Evidence for the formation of complex homopolymers in yeast. Both TIR-domain-containing adaptors TIRAP and TRAM attach to the inner leaflet of the plasma membrane when co-expressed in yeast, but the latter develops long curly filaments, although only in cells that do not express TIRAP [112]. The middle section and full Z-stack of a confocal set of images is shown (upper left panel). MyD88 forms thick spots in yeast cells, suggesting self-aggregation [112]. Confocal images superimposed to the indicated fluorescent channels are shown (lower left panel). The right panel shows equivalent transmitted light and fluorescence microscopy images of yeast cells expressing the indicated fusions of human SMOC proteins to either green or red fluorescent proteins (our own unpublished data). Scale bars depict 5 µm. (**b**) Scheme of the Sup35-based prion assay in yeast used to test MAVS and ASC prionic behavior [116]. When the candidate domain does not behave as a prion, Sup35 acts as a terminator factor and *ade1* mRNA is not translated properly. Thus, a colored adenine precursor accumulates leading to pink colonies (left). When the candidate protein domain, such as ASC^PYD^, shows a prionic-like behavior, Sup35 oligomerizes, and *ade1* translation is successful (right). This is phenotypically detected as a color change to white in yeast colonies due to proper adenine synthesis (adapted from Alberti et al. [119]).

We have observed that the overproduction of SMOC components in yeast leads to their aggregation as spots or filaments, evidenced by fluorescence microscopy, when they are fused to green or red fluorescent proteins. For example, TIR-domain-containing adaptor proteins form compact defined spots, like MyD88, or long filaments, like TRAM [112] (Figure 5a). The DD-containing DISC components, namely the Fas receptor and the FADD adaptor, also form filamentous structures in yeast, whereas CARD-containing ASC forms “speck”-like cytoplasmic spots reminiscent of those described in mammalian cells [113]. Moreover, the mitochondrial antiviral signaling (MAVS) protein, the core component of the riggosome SMOC in RIG-I-like receptor (RLR)-mediated signaling, forms a filament network (Figure 5a). This behavior is often disrupted when interactors are co-expressed (our unpublished data), substantiating that budding yeast can be used to study the hierarchy of assembly and the behavior of DD homotypic interactions of SMOC adaptor proteins in vivo.

Interestingly, *S. cerevisiae* has been used to prove that some of these adaptors show prionic behavior. Yeast has its own prion-like proteins with a physiological role; in fact, it codes for at least 24 proteins with a prion-forming domain (PrD) [114,115]. Cai et al. employed a yeast-based system to demonstrate the prion-like behavior of the riggosome MAVS and inflammasome ASC adaptor proteins, exchanging the Sup35 PrD for the DDs of these adaptors, either the CARD domain of MAVS or the PYD of ASC [116]. Both MAVS and ASC DDs are essential to oligomerize and trigger their downstream signaling pathways. When Sup35 PrD was exchanged for either of these domains, prionic behavior was preserved (Figure 5b). Reciprocally, both MAVS CARD and ASC PYDs could be replaced with yeast Sup35 PrD in mammalian cells, achieving an activation of innate immune signaling. Thus, both ASC- and MAVS-based prion-like filamentous platforms provided an appropriate environment for optimal caspase-1 and tank-binding kinase 1 (TBK1) or IκB kinase-ε (IKKε) activation, respectively [117,118].

*S. cerevisiae* is not the only fungal species in which prions have been found. The multicellular fungus *Podospora anserina* also possesses prion-like proteins whose gain-of-function state induces cell death in a way that resembles NLRP3 inflammasome signaling [120]. Prion-like polymerization likely represents an ancient strategy for signal transduction that has been retained and adapted in several organisms. This mechanism has been conserved from fungi to human cells, which makes the yeast model an appropriate system to study evolutionary insights of prion-like signaling in human inflammatory pathways [116].

## 5. Caveats and Challenges of SIGNALING by Cooperative Assembly Formation (SCAF) Yeast-Based Models

SMOCs are fundamental signaling hubs for the innate immune system, as they amplify and coordinate an appropriate response to ensure organism survival in higher eukaryotes [3,4]. Obviously, *S. cerevisiae* lacks an immune system, but its intracellular environment is a good minimal in vivo setting for studying SMOCs’ components isolated from their molecular milieu. Heterologous expression in yeast has demonstrated the possibilities that it offers countless times, both on an industrial level in terms of the production of proteins or chemical compounds (such as insulin, artemisinin, etc.) and on a research level in terms of functional and structure–activity protein studies [121,122,123]. The reconstruction of SMOC-based signaling pathways within yeast cells is a major challenge, but it can provide a ready and affordable alternative to elucidate interactions among their components and with the cellular landscape, as well as other multiple aspects of their biology. Ultimately, they could provide easy bioassays for the pharmacological screening for SMOC assembly inhibitors. Such yeast-based platforms would offer an in vivo setting for drug discovery with the advantage over mammalian systems that they are more cost-effective and easily manipulatable.

Nevertheless, the yeast model has its own difficulties in this field. One of the most notorious is inadequate post-translational modifications (PTMs) of heterologous proteins. Signaling by SMOCs usually depends on PTMs, such as palmitoylation, sumoylation, phosphorylation, or K63-linked ubiquitination [57,124,125,126,127], that may not spontaneously occur in yeast. Nevertheless, some of these PTMs can be recapitulated in yeast thanks to the presence of orthologous enzymes that take over the role of those in higher cells. This has been described in tau hyperphosphorylation in a yeast-based Alzheimer’s Disease model, where endogenous protein kinases carry out phosphorylation events essential to reproduce both tau’s pathological behavior and its negative regulation [128,129]. Also, in the case that yeast lacks ortholog enzymes required to carry out essential PTMs, the appropriate heterologous enzymes could be co-expressed. For example, glycosylation in yeast differs from that of humans in the hypermannosylation of the core glycan. In humans, these glycan structures are more complex, composed of N-acetylglucosamine, galactose, sialic acid, or fucose [130]. By deleting yeast mannosyltransferases and introducing a cassette with the human enzymes by homologous recombination, it has been possible to humanize this PTM in yeast [131].

In any event, yeast-based models aiming to support studies on the assembly of SMOCs or their readouts, such as caspase activity, must rely on easily detectable and/or quantifiable outputs, either based on growth recovery, the activity of reporter enzymes, or fluorescent reporters. Such platforms could be applied to (i) in vivo screens for the inhibitors or modulators of SCAF signaling in innate immunity or RCD; (ii) comprehensive mutagenic analyses, either random or directed, to assess the function of mutations found in the clinics, or simply to test structure–function relationships; the identification of caspase protein substrates, such as GSDMs, and novel SMOC interactors. Synthetic biology platforms may allow new strategies that will bring SMOC yeast models onto a new stage in the near future.

## 6. Conclusions

RCD pathways like apoptosis, pyroptosis, and necroptosis, among others, are crucial for the maintenance of tissue homeostasis and innate immunity. Therefore, their dysfunction is related to a wide range of pathologies, including inflammatory, autoimmune, and degenerative diseases, as well as cancer. The assembly of SMOCs, like the inflammasome, is a common feature of many RCD pathways, acting as platforms for the recruitment and activation of signaling molecules. The field demands additional experimental models to assess the function of these important druggable targets.

The budding yeast *S. cerevisiae*, an easy-to-manipulate, inexpensive unicellular model, provides an attractive experimental setting for the study of RCD-related signalosomes. Throughout the years, the heterologous expression of mammalian signaling pathways in yeast has steadily supported advances in the field. Issues like the specificity of the different adaptors to their effectors in these signaling complexes or the prion-like behavior of some of their components have been successfully addressed in this model. The implementation of synthetic biology strategies, together with the study of how RCD signaling proteins interact with yeast endogenous pathways, will surely contribute to developing improved humanized yeast models in the future.

## Figures and Tables

**Table 1:** Caspase inhibitors tested in *S. cerevisiae*.

Caspase ^1^	Inhibitor	Reference
Dm DCP-1	DIAP1	[82]
	Baculovirus p35 protein	[88]
	Baculovirus p49 protein	[88]
Dm DRONC	DIAP1	[89]
	Baculovirus p49 protein	[88]
Dm drICE	Baculovirus p35 protein	[88]
	Baculovirus p49 protein	[88]
	DIAP1	[88]
Ce CED-4	Ce CED-9	[90]
Hs CASP-8	Baculovirus p35 protein	[81,88]
	Cowpox virus CrmA	[91]
Hs CASP-9	XIAP	[92]
Hs CASP-3	Cowpox virus CrmA-mut	[83]
	XIAP, c-IAP1, c-IAP2	[93,94]
	Baculovirus p35 protein	[88]
	Baculovirus p49 protein	[88]
	ZVAD-fluoromethyl ketone	[83]
	Q-VD-OPh	[91]
	Ac-DEVD-chloromethyl ketone	[95]
	Aspartic vinyl sulphones	[95]
Hs CASP-7	Baculovirus p35 protein	[88]
	Baculovirus p49 protein	[88]
	XIAP	[88]
Hs CASP-2	Baculovirus p35 protein	[88]
	Baculovirus p49 protein	[88]
Hs CASP-4	Baculovirus p35 protein	[88]
Hs CASP-5	Baculovirus p35 protein	[88]
Hs CASP-1	Q-VD-OPh	[91]

^1^ Dm, Drosophila melanogaster; Ce, Caenorhabditis elegans; Hs, homo sapiens. CASP, caspase.

## Data Availability

Not applicable.
